# A Rare Case of Primary Duodenal Plasmacytoma: An Incidental Finding

**DOI:** 10.7759/cureus.37342

**Published:** 2023-04-09

**Authors:** Islam Mohamed, Mariam Haji, Noor Hassan, Hana Qasim, Rawan Rajab, Rami Rajab, Ali Ibrahim, Mir Zulqarnain, Esmat Z Sadeddin, Hassan Ghoz, Sobrina Mohammed

**Affiliations:** 1 Internal Medicine, University of Missouri Kansas City School of Medicine, Kansas City, USA; 2 Biology, Saint Louis University, Saint Louis, USA; 3 Gastroenterology and Hepatology, University of Missouri Kansas City School of Medicine, Kansas City, USA

**Keywords:** solitary extramedullary plasmacytoma, multiple myeloma, plasma cell neoplasm, gastrointestinal bleed, extramedullary plasmacytoma

## Abstract

Solitary extramedullary plasmacytoma (SEP) is a rare tumor due to the monoclonal proliferation of plasma cells without bone marrow involvement. Plasmacytomas are frequently encountered in bone or soft tissue but rarely occur in the gastrointestinal (GI) tract. They can present with a multitude of symptoms depending on their site. This report describes a case of SEP diagnosed as a duodenal ulcer (DU) during esophagogastroduodenoscopy (EGD) for iron deficiency anemia.

## Introduction

Solitary plasmacytoma is an uncommon plasma cell dyscrasia. Diagnosis is made by histology which confirms monoclonal plasma cell infiltration after excluding bone marrow involvement and myeloma-defining features [1]. Solitary plasmacytomas that present outside the bone and in soft tissue are commonly known as solitary extra-medullary plasmacytoma (SEP). Extramedullary plasmacytomas are rare with an incidence of 3-5% of plasma cell dyscrasias [2]. They most commonly arise from the head, neck, and upper respiratory tract. Solitary gastrointestinal (GI) plasmacytomas are even more unique, accounting for approximately 4% of extramedullary plasmacytomas [3]. Presenting symptoms depend on the location of the mass with abdominal pain, fatigue, GI bleeds, and altered bowel habits being the most reported for GI plasmacytomas in the literature [3]. We describe an atypical presentation of solitary duodenal plasmacytoma that was diagnosed via an upper GI endoscopy as part of the evaluation of iron deficiency anemia.

## Case presentation

**Figure 1:**
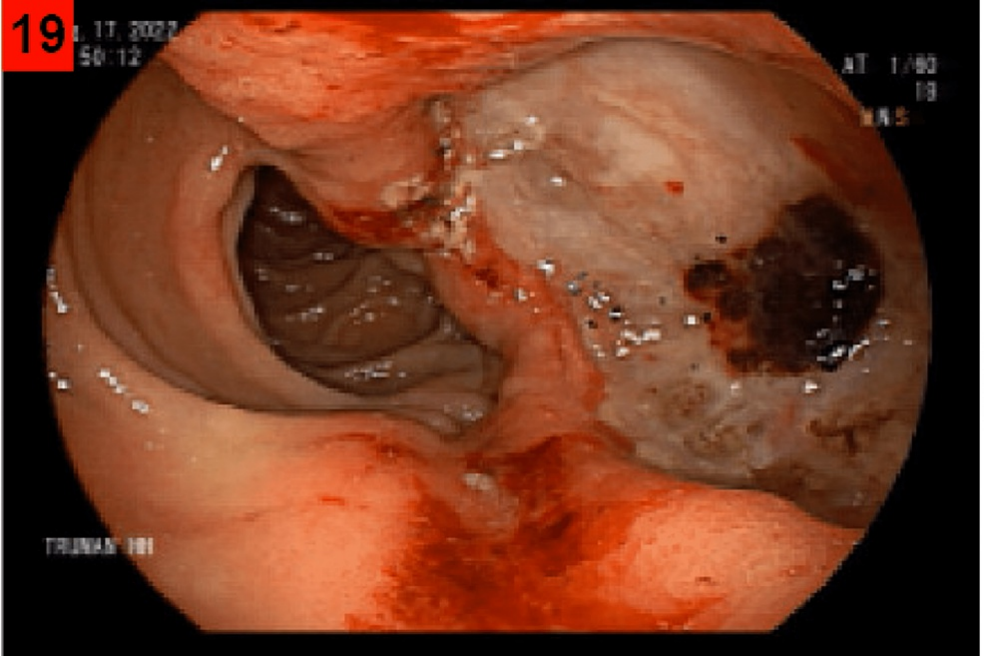
Large deformed bulging ulcer with deep base and overlying clot with active oozing.

**Figure 2:**
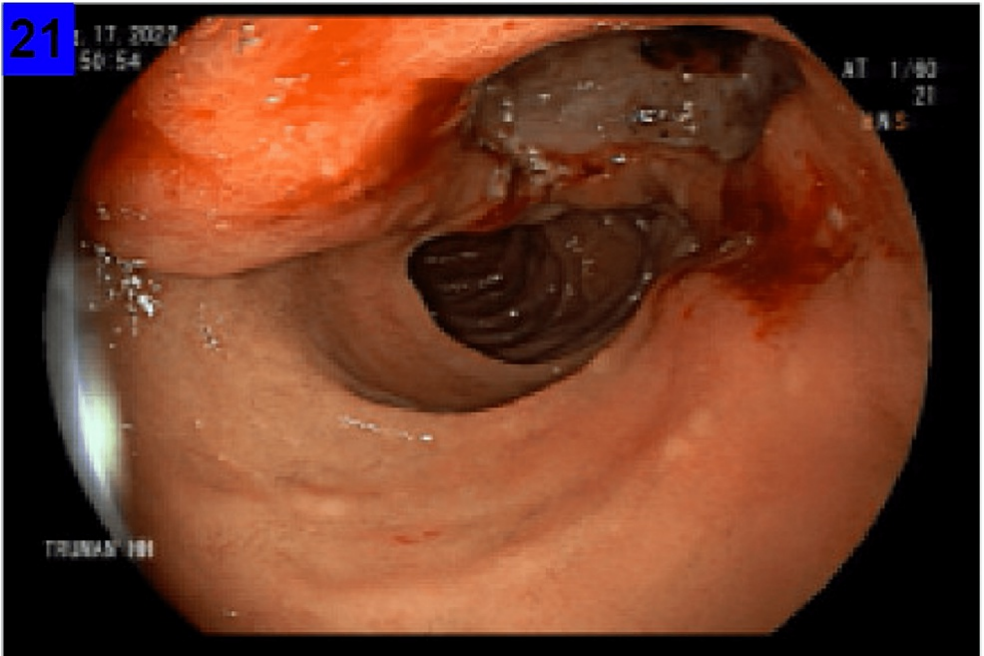
Biopsy from the ulcer base showed a picture of peptic duodenitis.

A 67-year-old male with a past medical history significant for prostate cancer status-post prostatectomy and chronic microcytic hypochromic anemia was referred to the gastroenterology clinic for further workup of Iron deficiency anemia. According to the patient, he was diagnosed with iron deficiency ten years ago and was prescribed iron supplements without knowing the exact cause of his anemia. He has recently reported fatigue and shortness of breath when exerting himself but otherwise has denied experiencing any gastroenterological symptoms including nausea, vomiting, dyspepsia, abdominal pain, diarrhea, or hematochezia. He denied melanotic stools but did have chronic black stools secondary to iron supplementation. Laboratory analysis revealed a hemoglobin level of 9.5 g/dl, a ferritin level of 44.2 ng/ml, and a transferrin saturation of 9%. He subsequently had an outpatient esophagogastroduodenoscopy (EGD) that revealed a large deformed, bulging, bleeding duodenal ulcer with a deep base (Figures 1, 2). Biopsies taken for the ulcer base later showed a picture of peptic duodenitis and were negative for Helicobacter pylori (H. pylori). 

**Figure 3:**
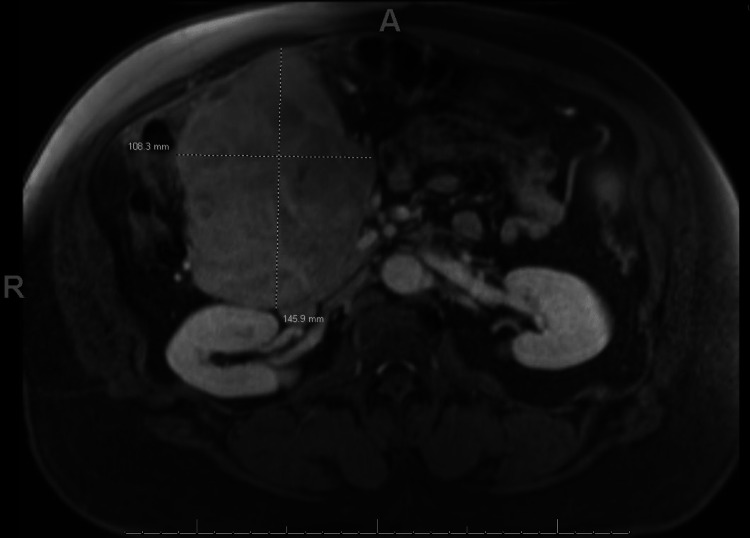
MRI abdomen/pelvis w/wo contrast showing a large heterogeneously enhancing mass in the second portion of the duodenum measuring 14.6 x 10.8 x 12.4 cm.

**Figure 4:**
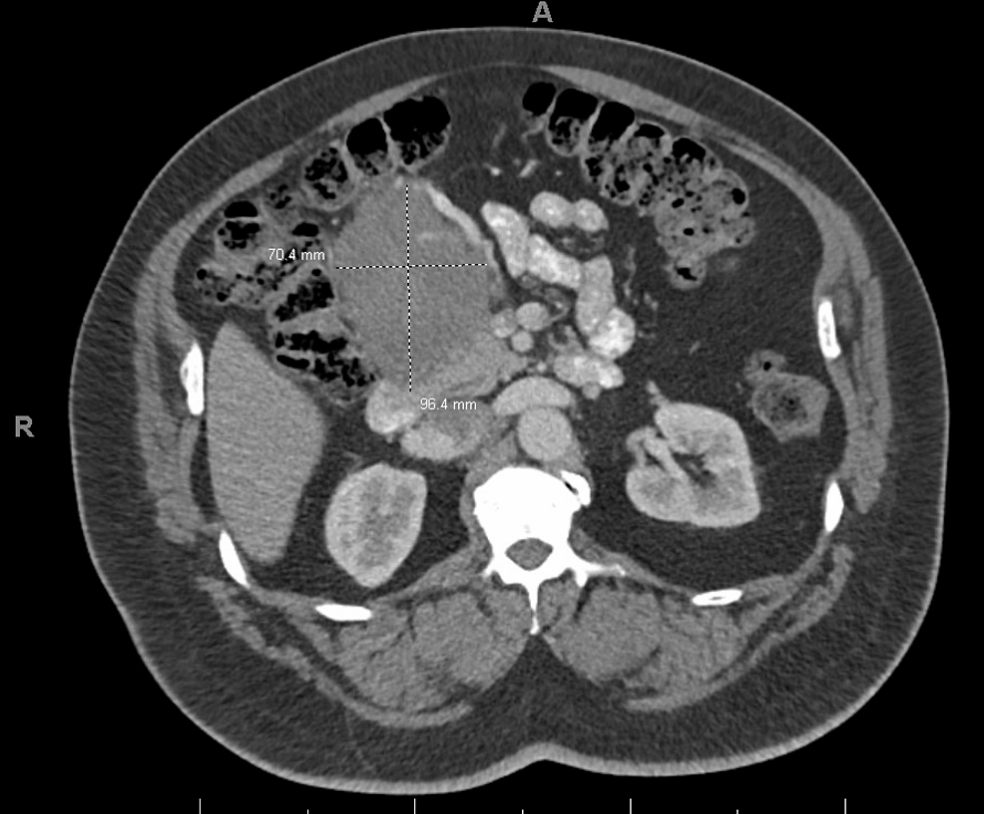
CT abdomen and pelvis following chemotherapy shows a reduction in the size of the duodenal mass to 9.6 x 7.0 x 7.2 cm.

Although the patient was vitally stable, he was admitted to the hospital for further workup and hemodynamic monitoring. Medical management constituted intravenous pantoprazole twice daily, intravenous fluids, and iron sucrose infusions. CT scan of the abdomen and pelvis revealed a large hyper-dense mass about the second portion of the duodenum adjacent to the duodenal ulcer, with neck measurements up to 2.2 cm. There were also visualized periaortic and peri-gastric lymph node enlargements measuring up to 1.9 cm. MRI of the abdomen and pelvis was subsequently obtained which showed a large, heterogeneously enhancing restricted mass about the second portion of the duodenum with restricted diffusion, measuring 14.6 x 10.8 x 12.4 cm (Figure 3). The patient underwent a biopsy of the abdominal mass by interventional radiology which later revealed a plasma cell neoplasm. Bone marrow biopsy revealed no morphologic, immunophenotypic, or cytogenetic evidence of plasma cell myeloma. No chromosomal abnormalities were detected by Fluorescence in situ hybridization (FISH). The serum protein electrophoresis with immunofixation revealed a noteworthy monoclonal IgA lambda band, measuring 0.5 g/dL, which is abnormal as compared to the normal range of 0 g/dL. Similarly, the urine protein electrophoresis showed the presence of monoclonal lambda light chain, measuring approximately 6.1 mg/dL, which is also outside the normal range of 0 mg/dL. Light chain assays showed lambda light chains of 153 mg/L (normal 5.70-26.30 mg/dl), kappa light chains of 10.45 mg/L (normal 3.30-19.40 mg/dl), with a ratio of 0.07 mg/dl (normal 0.26-1.65 mg/dl). A positron emission tomography (PET) scan showed ​​a bulky hypermetabolic duodenal mass with a metastatic left periaortic lymph node; no active uptake was noted elsewhere. A multidisciplinary tumor board recommended surgical evaluation followed by radiation therapy. A surgical evaluation of the patient recommended a Whipple procedure for the excision of the mass, which the patient rejected. Following discussions with multiple myeloma specialists and reviewing the limited literature on gastrointestinal solitary extra-medullary plasmacytoma (GI SEP), he was prescribed Bortezomib, Lenalidomide, and Dexamethasone for four cycles. His follow-up CT scan revealed a significant reduction in the size of the duodenal mass to 9.6 x 7.0 x 7.2 cm from 14.6 x 10.8 x 12.4 cm, normalization of Lambda chains, and absence of monoclonal bands (Figure 4). Currently, the hematology clinic is monitoring the patient, with the aim of performing an autologous bone marrow transplant and local resection of the tumor.

## Discussion

Plasma cell neoplasms are a group of entities characterized by the clonal proliferation of plasma cells. They vary in their presentation and clinical effects and include plasma cell myeloma, plasma cell leukemia, plasmacytoma, and monoclonal immunoglobulin deposition disease. SEP is extremely rare and has an incidence of 0.04 cases per 100,000 individuals around the world [4]. The presentation of solitary plasmacytoma depends on the involved organ, and as extramedullary plasmacytoma doesn’t involve bone or bone marrow, the typical findings of multiple myeloma including anemia, hypercalcemia, bone pain, and renal insufficiency are typically absent, which makes the diagnosis more challenging [5]. The reported cases in the literature were for symptomatic patients who presented with abdominal pain, vomiting, and upper GI bleeding with imaging and endoscopic findings of duodenal mass [6,7]. To our knowledge, only two cases of plasmacytoma presented with a duodenal ulcer were reported in the literature [8,9], which implies the extremely rare presentation of our patient.

As expected, patients with duodenal plasmacytoma like other duodenal tumors may remain asymptomatic until there is significant tumor growth. Symptoms may include abdominal pain, nausea, weight loss, intestinal obstruction, and GI bleeding [3]. In our patient, it was surprising that he was completely asymptomatic despite a significantly large and bleeding duodenal ulcer, with the only lab finding being iron deficiency anemia; this highlights the importance of investigating GI malignancies as the first possible cause of anemia in adult males and post-menopausal females.

In the Western world, 10% of the population have suffered from duodenal ulcers (DU) at some point in their life [10]. These ulcers occur when a mucosal break extends through the muscularis mucosa into the deeper layers of the intestine [11]. DU can be asymptomatic or present with typical complaints of epigastric pain, nausea, or ulcer-related complications like bleeding, perforation, or obstruction [12]. Although duodenal ulcers are almost always benign and taking biopsies in benign-looking ulcers is not recommended, endoscopically suspicious ulcers must be biopsied [13]. Additionally, suspicious masses found during EGD should also be followed by appropriate imaging in order to characterize tumors and rule out metastasis [14].

The treatment of choice for extramedullary plasmacytoma is debatable due to the limited cases in the literature and the absence of prospective studies to assess outcomes. Wen et al compared different treatment modalities including radiotherapy, surgical resection, and chemotherapy in a retrospective study that included 55 patients with a pathologically confirmed diagnosis of SEP [15]. It concluded that radiotherapy is the treatment of choice for SEP with better local recurrence-free survival (LRFS), multiple myeloma-free survival (MMFS), progression-free survival (PFS), and overall survival (OS). The European Expert Panel has suggested that radiotherapy be administered at a dose of 40-50 Gy, delivered over a period of approximately 4 weeks, as it poses a low risk of relapse [16-17]. If a complete surgical resection was attempted at the time of diagnosis, the role of adjuvant radiotherapy remains unclear and is decided on a case-to-case basis depending on suspicion of local recurrence [18-19]. If incomplete resection was attempted, the use of local radiotherapy is favorable to surgery, chemotherapy, or observation [18-19]. Due to the rarity of duodenal plasmacytoma and the large majority of studies assessing head and neck SEP and not GI SEP, expert opinions by surgery and oncology will determine the best treatment option for the patient. 

## Conclusions

When treating patients with iron deficiency anemia, a high index of suspicion should be maintained for the presence of occult malignancies. SEP is a rare tumor with a variety of clinical presentations. It can rarely present in the GI tract with manifestations of iron deficiency anemia in the absence of myeloma-defining features. In confirmed cases, a multidisciplinary approach should be used in order to determine the most appropriate course of treatment.
